# Compassion fatigue, burnout and compassion satisfaction among family physicians in the Negev area - a cross-sectional study

**DOI:** 10.1186/2045-4015-2-31

**Published:** 2013-08-15

**Authors:** Nurit El-bar, Amalia Levy, Hedy S Wald, Aya Biderman

**Affiliations:** 1 Ness Ziona Mental Health Center, Ness Ziona, Israel; 2 Department of Epidemiology, Faculty of Health Sciences, Ben Gurion University of the Negev, Beer Sheva, Israel; 3 Department of Family Medicine, Warren Alpert Medical School of Brown University, Providence, RI, USA; 4 Department of Family Medicine and Siaal Research Center for Family Medicine and Primary Care, Division of Community Health, Faculty of Health Sciences, Ben Gurion University of the Negev, Beer Sheva, Israel

**Keywords:** Burnout, Compassion fatigue, Compassion satisfaction, Family practice, Secondary post-traumatic stress syndrome

## Abstract

**Background:**

Compassion fatigue among health care professionals has gained interest over the past decade. Compassion fatigue, as well as burnout, has been associated with depersonalization and suboptimal patient care. Professional caregivers in general are exposed to the risk of compassion fatigue (CF), burnout (BO) and low levels of compassion satisfaction (CS). While CF has been studied in health care professionals, few publications address its incidence among family physicians, specifically. The objectives of this study were to assess the prevalence and severity of CF among family practitioners (FPs) in the Negev (Israel’s southern region), evaluating the correlations between CF, BO and CS and their relations with socio-demographic variables and work related characteristics.

**Methods:**

Self-report anonymous Compassion Satisfaction and Fatigue Test questionnaires (CSFT) measuring CF, BO, and CS were distributed among 194 family physicians at Clalit Health Services clinics in the Negev between July 2007 and April 2008. Correlations between CF, BO and CS were assessed. Multivariable logistic regression models with backward elimination were constructed.

**Results:**

128 (66%) physicians responded. 46.1% of respondents scored extremely high and high for CF, 21.1% scored low for CS and 9.4% scored high for BO. Strong correlations were found between BO and CF (r = 0.769, p < 0.001), and between BO and CS (r = −0.241, p = 0.006), but no correlation was found between CS and CF. The logistic regression model showed that the only factor associated with a significantly increased risk for CF was former immigration to Israel. Increased risk for BO was associated with female gender, history of personal trauma and lack of academic affiliation. Higher CS was associated with holding management positions and teaching residents.

**Conclusions and policy recommendations:**

Family physicians in the Negev are at high risk for CF, with the potential for CF- associated patient dissatisfaction, compromised patient safety and increased medical error. We propose creation of a CF educational and early intervention treatment program for family physicians and other health care professionals. Such programs would train facilitators of physician well-being and resiliency building. We also recommend analyzing contributing variables and organizational factors related to higher CF. Policy recommendations include integrating such programs within required risk management continuing medical education.

## Background

The physical and emotional impacts of caring within often stress-filled health care environments are gaining increasing attention [1]. Terms such as compassion fatigue [2], burnout [3], and secondary traumatic stress [4] have been used to describe conditions resulting from being continuously subjected to highly stressful circumstances in a professional capacity [5]. Given that these concepts are closely related [6] in describing negative effects of an individual’s work life quality [1], there can be ambiguity in definitions, with, for example, the terms secondary stress and compassion fatigue often used interchangeably in the literature [4,7,8]. While compassion fatigue had been previously described as a form of burnout [9], recent literature argues that these terms (as well as secondary traumatic stress) reflect related, though distinctly unique concepts [1] and are not mutually exclusive. Compassion fatigue, for example, may coexist with burnout [6]. We concur with conceptualizations which differentiate these terms and provide the following working definitions as a foundation for our work:

Compassion Fatigue (CF) [2] is the negative aspect of our work as helpers, the profound emotional and physical exhaustion that helping professionals and caregivers can develop over time [4,10]. CF is also called secondary traumatic stress disorder (STSD), among professional caregivers who care for patients with post-traumatic stress disorder (PTSD) and “suffering patients” in general [4,11-16].

Within the health professions, burnout- BO has been defined as an experience of physical, emotional, and mental exhaustion, caused by long-term involvement in situations that are emotionally demanding [17].

While there is some similarity between BO and CF symptomatology, significant differences exist. Burnout may affect any worker, and describes the powerlessness of those who have low work satisfaction, mainly due to demands that are beyond the capacity of the worker, a phenomenon in which a previously committed worker disengages from his/her work in response to excessive and prolonged work-related stress. CF, by contrast, is typical to the caring professionals: it may occur as a result of a single exposure to a traumatized patient [18,19]. It can emerge suddenly and recovery is typically faster [4].

In contrast, compassion satisfaction-CS is the sense of pleasure derived from helping others, and the degree of support received from colleagues [20].

Compassion fatigue, as well as burnout, has been associated with depersonalization and suboptimal patient care [9].

Symptoms of CF include: 1) intrusive thoughts or images and feelings of distress or physiological reactivity to reminders of the patient’s traumatic experience; 2) avoidance and numbness; 3) hyper arousal and 4) exhaustion and fatigue [4,9,21].

Risk factors for developing CF include working with patients who have experienced traumatic events, one’s own history of traumatic events, having extended relationships with patients, lack of support systems, lack of experience, imbalance between work and personal life and absence of self-awareness [10,11].

Potential protecting factors include adequate social support, self and occupational development, and self-awareness [4,7,9,10,12,14-16,22,23]. The professional caregiver with CF may experience psychological distress, cognitive shifts, and relational disturbances [20]. In addition, CF may be associated with decreased productivity, increased sick leaves and higher turnover rates [12,13,23].

Professional caregivers in general are exposed to the risk of compassion fatigue (CF), burnout (BO) and low levels of compassion satisfaction (CS).

CF is highly prevalent among the helping professionals with, for example, 10% of trauma counselors [22], and 49% of professionals working in child protection found to be at high to extremely high risk [24]. Within a sample of social workers, 15.2% reported having the three criteria of STSD - Secondary Traumatic Stress Disorder [18] and approximately 26% of hospice nurses (within 22 hospice facilities) were in the high risk category [11].

Family physicians may be more prone to developing compassion fatigue due to some characteristics of their professional work. Such characteristics include ongoing involvement with relatively difficult patient care encounters including complexity within chronic illnesses, physician-patient relationships characterized by emotional involvement over long periods, typical lack of professional supervision, and heavy daily patient care load.

In line with this, Woolhouse and colleagues used a qualitative methodology to assess a group of inner city family physicians and found that these physicians were experiencing vicarious traumatization [25].

While characteristics of CF have been studied in health care professionals, few publications address its incidence among family physicians specifically. The objectives of this study were to assess the prevalence and severity of CF among family practitioners (FPs) in the Negev. Additional objectives included evaluating the correlations between CF, BO and CS, and their relations with socio-demographic variables and work related characteristics.

## Methods

Between July 2007 and April 2008, anonymous Compassion Satisfaction and Fatigue Test questionnaires (CSFT) were distributed to all FPs in Clalit Health Services in the Negev region, n = 194 (135 board certified and 59 Family Medicine residents). Demographic, practice and personal data were collected within the questionnaires. Some questionnaires were sent by mail and others delivered directly during residents’ CME courses or staff meetings.

The CSFT is a 66 item self- report questionnaire comprised of three subscales, i.e. CF, BO and CS [20]. Each question is scored on a Likert scale of 0 to 5 (0 as lack of CF/BO symptomatolgy, 5 as most often) The scores for the questions are summed for each of the three subscales. We conducted a pilot study of this questionnaire which provided good evidence for reliability with internal consistency alphas of the three subscales as follows – CF (0.87), BO (0.9) and CS (0.87) [19,20]. The questionnaire was translated and validated in Hebrew; the translation process was then cross-checked by cross translation [26]. Additional background information was gathered from participants. In addition to the two questions of the CSFT assessing personal trauma in their childhood and/or as an adult, participants were queried about their involvement in management positions, academic activities (teaching students or residents and/or research), academic affiliation (none, instructor or higher level), employment in other clinics and about their hobbies. These questions were added as possible contributing factors to CF, BO and CS.

Missing data (0.4% of items from the CSFT) was completed by calculating the average score of the remaining respondent’s scores for that specific dimension. Information on age, gender, marital status, country of birth and specialization of the FPs who did not return the questionnaires was collected from the Department of Family Medicine database at Ben-Gurion University of the Negev.

Subscale scores were initially interpreted according to theoretically derived scale values ranging from extremely low risk to extremely high risk for experiencing CF, BO and from low to high potential for CS [20]. In order to estimate adjusted risk for CF, BO and CS, each scale was divided into two groups, a group at any risk and a group without risk. In the CF and BO subscales, the group “at risk” included –‘extremely high risk’, ‘high risk’ and ‘moderate risk’. In the CS subscale, the group defined as “at risk” included those who scored ‘extremely low’, ‘low’ and ‘moderate’ on the compassion satisfaction scale.

Statistical analysis was performed using SPSS package (SPSS, Chicago, IL version 17) Correlation between CF, BO, and CS was calculated using Pearson’s correlation. Statistical significance was calculated using the chi-square test and Fisher’s exact test for differences in qualitative variables and t-test or one way ANOVA for differences in continuous variables. Multivariable logistic regression models with backward elimination were constructed for confound control. Odds ratios (OR) and their 95% confidence interval (CI) were computed. In all analyses, p < 0.05 was considered statistically significant.

## Results

Of 194 questionnaires distributed, 128 were returned (66% response rate). Response rates were higher among residents (N = 54, 91.5% vs. 54.1%, p < 0.001) and Israeli born physicians (84.2% vs. 70.6% from former Soviet Union, p = 0.016). The respondents’ mean age was 43.6 ±8.23 years, and 65 were female (52.4%). Most physicians were married (N = 109, 87.2%), born abroad (N = 93, 74.4%) and studied medicine abroad (N = 99, 79.2%). More than half were board certified FPs (N = 72, 57.1%), involved in teaching medical students (N = 88, 76.5%) but had a low academic affiliation or no academic affiliation at all (N = 101, 90.2%). Management positions were held by 44 physicians (25%). The respondents had a mean of 15.4 ± 9.66 years of medical experience. On average, study participants had 1,390 ± 557 listed patients and worked in clinics with other physicians (4.5 ± 2.8 on average). The primary workplace for most was an urban clinic (N = 95, 78.5%).

**Figure 1:**
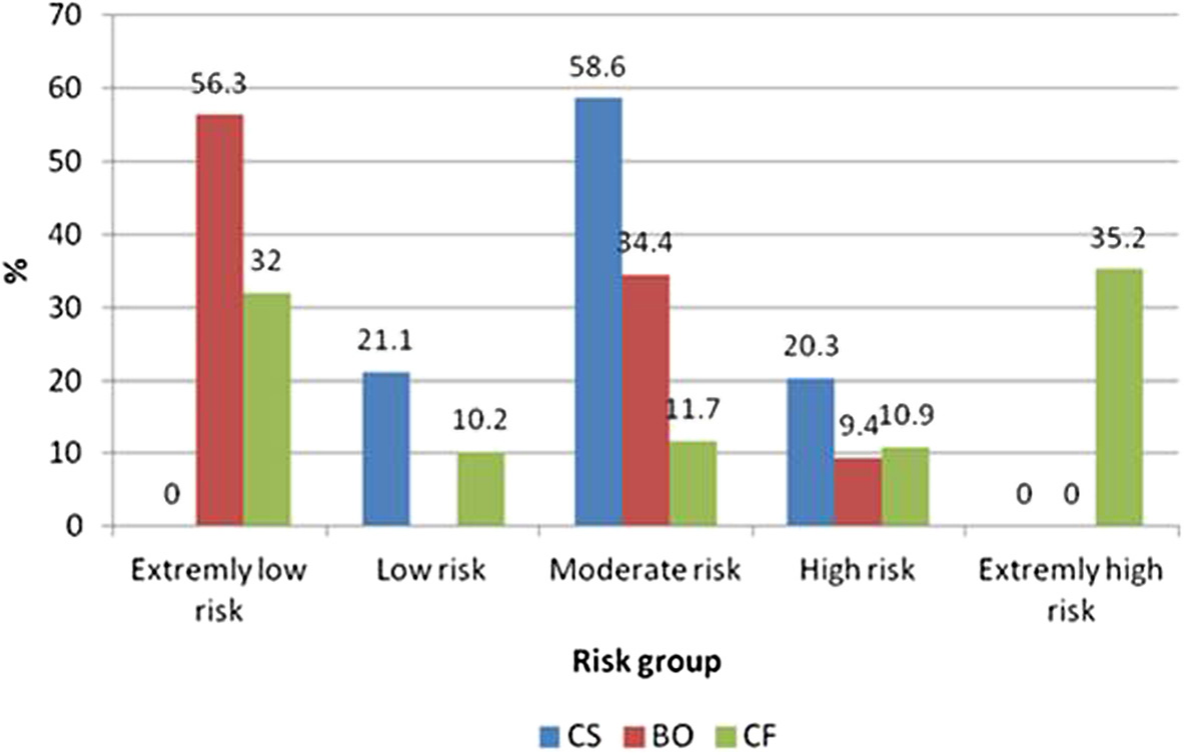
**Describes the distribution of respondents by percentage in five risk groups for CF and CS and in four groups for BO, scaling from extremely low to extremely high.** CF=compassion fatigue, BO=burnout and CS=compassion satisfaction.

Figure 1 depicts the distribution of respondents by percentages of CF, BO and CS according to their severity level. Findings indicate that 35.2% of FPs are at extremely high risk for CF, 9.4% are at high risk for BO and 21.1% are at risk for low CS.

We found a strong positive correlation (r = 0.769, p < 0.001) between BO and CF, and a negative correlation (r = −0.241, p = 0.006) between BO and CS. No correlation was found between CF and CS.

Table 1 lists the prevalence of categorical variables in dichotomous groups (a group at risk and a group without risk) regarding CF, BO and CS. Being born abroad and having a low / no academic affiliation are associated with increased risk for CF (p = 0.032 and 0.009 respectively). No differences were found for all other variables (gender, marital status, country of medical study, specialization, location of primary or secondary clinics, management position, teaching students or residents and academic activity). Factors which were associated with high levels of BO were female gender (p = 0.024), being born abroad (p = 0.012), studying medicine abroad (p = 0.004), having a low/no academic affiliation (p = 0.002), and experiencing personal trauma in the past (p = 0.004).

**Table 1:** Comparison of the risk for compassion fatigue (CF), burnout (BO) and compassion satisfaction (CS) by socio-demographic, professional and other background variables

**Variables**		**N**	**Group without risk**	**Group with risk**	**P value**
**Prevalence of CF (%)**					
Country of birth	Israel	32	59.4	40.6	0.032
	Others	93	37.6	62.4	
Acadmic affiliation	Instructor	49	36.7	63.3	0.009
	Lecturer and above	11	81.8	18.2	
**Prevalence of BO (%)**					
Gender	men	59	67.8	32.2	0.024
	Women	65	47.7	52.3	
Country of birth	Israel	32	75	25	0.012
	Other	93	49.5	50.5	
Country of medical Studies	Israel	26	80.8	19.2	0.004
	Other	99	49.5	50.5	
Academic affiliation	Instructor	49	51	49	0.002
	Lecturer and above	11	100	0	
Trauma in the past	Never	22	68.2	31.8	0.004
	A few times	76	63.2	36.8	
	Often	30	30	70	
**Prevalence of CS (%)**					
Country of birth	Israel	32	34.4	65.6	0.028
	Other	93	16.1	83.9	
Management position	Yes	28	46.4	53.6	<0.001
	No	84	9.5	90.5	
Teaching resident	Yes	35	37.1	62.9	0.001
	No	76	10.5	89.5	
Academic affiliation	Instructor	49	16.3	83.7	0.014
	Lecturer and above	11	54.5	45.5	

A low CS was associated with lack of academic affiliation ( p = 0.014) and being born abroad (p = 0.028). Involvement in management (p < 0.001) and teaching residents (p = 0.001), were associated with high levels of CS.

A separate analysis of the 135 board certified FPs revealed an association between low levels of BO and management positions (p = 0.021), teaching students (p = 0.012) and teaching residents (0.014) and between high levels of CS and involvement in research activity (p = 0.014).

**Figure 2:**
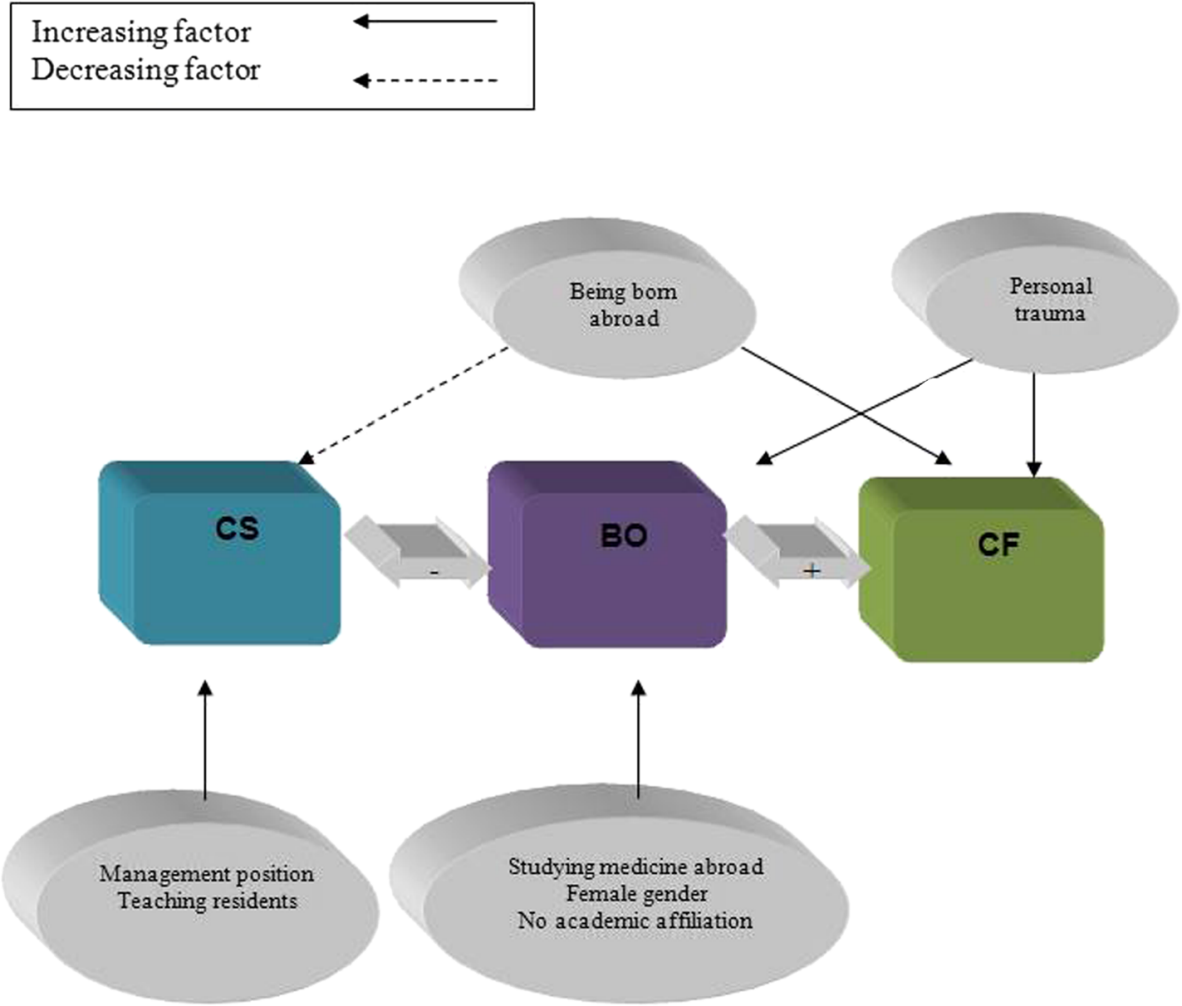
Correlation between compassion fatigue (CF), burnout (BO) and compassion satisfaction (CS) and their background variables.

Table 2 lists the logistic regression models of the odds ratios and the 95% confidence intervals for the three dimensions. The only factor associated with high levels of CF was being born abroad. Female gender, personal trauma, and lack of academic affiliation were associated with high levels of BO. Management positions and teaching residents were associated with high CS. The highest odds ratio for high CS was found for being involved in management activities (Figure 2).

**Table 2:** A multivariate logistic regression models with backward elimination for Compassion fatigue (CF) (n = 123), burnout (BO) (n = 122) and compassion satisfaction (CS) (n = 103)

	**Variables**		**.O.R**	**CI 95%**		**P value**
				**Lower**	**Upper**	
CF*	Country of birth	Other country vs. Israel	2.45	1.075	5.582	0.033
†BO	Gender	Female vs Male	2.423	1.034	5.676	0.042
	Country of medical studies	Other country vs. Israel	3.042	0.929	9.955	0.66
	Trauma	Yes vs. No	3.001	1.486	6.058	0.02
	Academic affiliation	No vs. Lecturer and above	3.822	1.106	13.364	0.034
	Academic affiliation	Instructor Vs. Lecturer and above	3.761	1.105	12.8	0.034
‡CS	Age		1.05	0.968	1.139	0.238
	Country of birth	Other country vs. Israel	1.637	0.485	5.524	0.427
	Management	No vs. Yes	5.596	1.634	19.165	0.006
	Teaching residents	No vs. Yes	4.139	1.07	16.007	0.04

*Variables included in the initial model of CF were: age, gender, country of birth and academic affiliation.

†Variables included in the initial model of BO were: age, gender, country of birth, country of medical studies, personal trauma and academic affiliation.

‡Variables included in the initial model of CS were: age, gender, country of birth, country of medical studies, management position, teaching residents, academic affiliation, and research activity.

*OR*, odds ratio, *CI*, confidence interval.

## Discussion

### 1) Statement of principal findings

We evaluated the risk for CF, BO and CS among FPs in the Negev region with a response rate of 66%. CF was most prevalent, with 46.1% of respondents scoring extremely high or high. Only 9.4% scored high for BO and 21.1% scored low for CS. We found a strong correlation between CF and BO but no correlation between CS and CF. Logistic regression models indicated that high compassion fatigue was associated with being born abroad. High risk for CS was associated with holding a management position and teaching residents. A sharp divide in risk for CF with distinct groups was a surprising finding. These two large groups included FPs at extremely high risk (35.2%) and at extremely low risk (32%). The possibility of group-specific underlying characteristics and relevance to CF, BO, and CS is a worthy topic for further research.

### 2) Strengths and weaknesses of the study

This study addresses the issue of CF among family physicians. The response rate was high, making the results of the study significant and valuable.

Limitations of our study include the sample consisting solely of FPs in the Negev area, with a relatively high percentage of physicians who have immigrated to Israel and who have no or low academic affiliation. This may have skewed the results towards higher levels of CF.

### 3) Meaning of the study

Several possible explanations can be offered for the high risk for CF in our study. In our sample, 74.4% of physicians immigrated to Israel, an experience that can be fraught with hardship and personal trauma [27,28]. This study was conducted in the Negev area, considered peripheral in the Israeli health care system. A recent study in Canada, found that the prevalence of probable post-traumatic stress disorder among physicians in a rural remote area was 4.4% [29].

Most of the respondents (90.2%) had a low academic affiliation or no affiliation at all. Given that foreign - born physicians and international medical graduates are typical in many countries, these findings are likely applicable and relevant within other countries as well. Another possible explanation for the finding of high level of CF is lack of awareness of this phenomenon and relative absence of intervention programs.

We found that a high level of CS is associated with low levels of BO, but CS was not associated with CF. In the literature, the association between CS and CF remains unclear. It may be that CF masks the ability to experience CS [21,23]. Another possible explanation is that CF is itself an acute reaction and thus not preventable by work satisfaction which is built over time. Work satisfaction may, however, be a means of defense for workers against BO.

We found a very high proportion of FPs at risk for CF. These findings are comparable to Conrad and colleagues’ findings [24] of 49.9% of child protection workers at high to very high risk for compassion fatigue. These levels are significantly higher than those found in studies investigating professionals in other fields, such as trauma counselors (10%) [29], social workers (15.2%) [18] and hospice nurses (26%) [11]. Family physicians’ longitudinal close relationships with their patients might account for these findings. We found that 9.4% of the physicians were at high risk for BO. This level of risk is consistent with other studies in the field such as the EGPRN study in Europe [17] which found 12% of FPs to be at high risk for BO. A 2001 study in Israel, using the BO construct, found that more than 59% of family physicians were in a very high burnout category, compared to about 15.5% in 1994 [30].

The high correlation between BO and CF (0.70) may support the interpretation of these as similar phenomena and thus merits further study.

The results of this study reveal the severe risk for CF among FPs in the Negev area, and calls for the immediate development and implementation of appropriate prevention and treatment programs. Untreated CF may negatively affect FP’s work-product and well-being, impacting patient safety.

### 4) Future research

Further studies should include cohorts of FPs from different regions and countries, and other medical specialties. Further research is required in order to determine other risk factors and best practices for physician well-being and resiliency promotion at individual and organizational levels. Evaluation of both educational programs for prevention and of treatment programs for CF is recommended.

The method for assessment of the CF, BO, and CS syndromes was recently revised to the shorter version Professional Quality of Life Questionnaire (ProQOL version 5 questionnaire) [31] and the use of this version within future research will be of interest. This shorter version was not validated in its Hebrew translation at the time of our study.

### 5) *Policy considerations*

Based on our findings, we propose that programming be implemented within the workplace to raise physicians’ and other health care professionals’ awareness of CF risk factors as well as the phenomena of CF and BO and associated symptoms. Ongoing wellness programs [32], continuing medical education programs which can include mindfulness training, reflective writing, and/or collegial support [33], and/or participation in Balint groups for initiating and maintaining professional networks [34] may be helpful interventions for targeting prevention and for building professional resiliency and well-being [35]. Such categories encompass factors such as work-life balance, attending to spiritual needs, obtaining regular professional supervision [20,34], initiating psychotherapy [4], overall good self-care (personal), awareness of goals, fostering professional networks (professional) and cultivating a comfortable workplace environment with a culture of support and respect (organizational) [16,36,37]. Programs run by health-care organizations in the UK, Canada, and the US to improve physician wellness include wellness educational activities, online resource materials, and/or free confidential services for physicians with mental or physical concerns or addictions [38]. In addition, CF treatment methods such as accelerated recovery programs [10,32,33,36] have been described. Conducting screening measures on a regular basis with support and facilitation of treatment as needed is also recommended.

In light of our presented findings and noted increasing concern among health care practitioners and health care organizations, the establishment of a national FP working subgroup on Physician Well-Being and Resiliency for addressing issues of CF and BO prevention appears warranted. In addition to exploring avenues for improvement in organizational policies and work environments, such a working subgroup could conceivably oversee creation, promotion, and implementation of programs within clinics/hospitals with training of facilitators and of therapists for treatment of CF and BO symptomatology. Such models have proven feasible with creation of self-sustaining centers for coping with CF manifestation [9]. Early intervention educational efforts within medical schools [39] with curricula components of self-care, fostering reflective self-awareness, and wellness programs for promoting personal and professional resiliency as integral to professionalism [40] may prove fruitful for preventing onset of CF and BO in residency and ultimately in professional practice.

## Conclusions

Family physicians in the Negev are at risk for CF. Untreated CF may negatively affect family physicians’ work-product and well-being and thus potentially compromise patient safety. The creation and implementation of preventive resiliency training programs is recommended as well as appropriate treatment interventions as needed. Given the potential for physicians’ experience of CF and/or BO symptomatology to negatively impact patient-centered care and compromise patient safety with increased medical error, we urge that self-care and CF and BO risk reduction and treatment interventions be regarded as ethical imperatives for a compassionate and competent health care provider.

### Ethical approval

The study received exemption by the local IRB.

## Competing interests

The authors declare that they have no competing interests.

## Authors’ contributions

All authors contributed to the design of the study. NE was responsible for writing the first draft. All authors analyzed and interpreted the data and participated in writing the final draft. All authors read and approved the final version of the manuscript.

## Authors’ information

NE, MD, is a resident in psychiatry at Ness Ziona mental health center, active in developing programs for the prevention and treatment of compassion fatigue among physicians. AL, PhD, is a professor and manager of the MPH program in the Public Health department, faculty of health sciences, Ben Gurion University of the Negev. HSW, PhD, is Clinical Associate Professor of Family Medicine at the Alpert Medical School at Brown University where she oversees the Family Medicine Clerkship reflective writing curriculum. AB, MD, is a professor of family medicine in the department of family medicine, division of community health, the faculty of health sciences, Ben Gurion University of the Negev.
